# Seabirds boost coral reef resilience

**DOI:** 10.1126/sciadv.adj0390

**Published:** 2023-12-06

**Authors:** Cassandra E. Benkwitt, Cecilia D’Angelo, Ruth E. Dunn, Rachel L. Gunn, Samuel Healing, M. Loreto Mardones, Joerg Wiedenmann, Shaun K. Wilson, Nicholas A. J. Graham

**Affiliations:** ^1^ Lancaster Environment Centre, Lancaster University, Lancaster LA1 4YQ, UK.; ^2^ Coral Reef Laboratory, School of Ocean and Earth Science, University of Southampton, Southampton SO143ZH, UK.; ^3^ The Lyell Centre, Heriot-Watt University, Edinburgh EH14 4AS, UK.; ^4^ Animal Evolutionary Ecology, Institute of Evolution and Ecology, University of Tübingen, Auf Der Morgenstelle 28, 72076 Tübingen, Germany.; ^5^ Australian Institute of Marine Science, Indian Ocean Marine Research Centre, Crawley, WA 6009, Australia.; ^6^ University of Western Australia, UWA Oceans Institute, Crawley, WA 6009, Australia.

## Abstract

Global climate change threatens tropical coral reefs, yet local management can influence resilience. While increasing anthropogenic nutrients reduce coral resistance and recovery, it is unknown how the loss, or restoration, of natural nutrient flows affects reef recovery. Here, we test how natural seabird-derived nutrient subsidies, which are threatened by invasive rats, influence the mechanisms and patterns of reef recovery following an extreme marine heatwave using multiyear field experiments, repeated surveys, and Bayesian modeling. Corals transplanted from rat to seabird islands quickly assimilated seabird-derived nutrients, fully acclimating to new nutrient conditions within 3 years. Increased seabird-derived nutrients, in turn, caused a doubling of coral growth rates both within individuals and across entire reefs. Seabirds were also associated with faster recovery time of *Acropora* coral cover (<4 years) and more dynamic recovery trajectories of entire benthic communities. We conclude that restoring seabird populations and associated nutrient pathways may foster greater coral reef resilience through enhanced growth and recovery rates of corals.

## Teaser

Seabirds provide nutrients to coral reefs that accelerate coral growth and recovery after an extreme marine heatwave.

## INTRODUCTION

Human-caused climate change is already causing extensive damage to ecosystems worldwide, with the impacts predicted to continue accumulating (*1*). While reducing greenhouse gas emissions is necessary to decrease the rate of warming and associated impacts, even with near-complete emissions reductions, warming of at least 1.5°C is likely locked-in for the coming decades (*1*, *2*). Therefore, it is also important to identify and promote local conditions that bolster species and ecosystem resilience to climate change in the near term.

Tropical coral reefs are one of the ecosystems most vulnerable to climate change, as increasing frequency and intensity of marine heatwaves causes mass die-offs of corals (*3*), which are the foundation species of these ecosystems (*4*). Even remote reefs are threatened by climate change, yet local conditions and communities produce spatial heterogeneity in resilience (*5*, *6*). Nutrients are one important driver of this variation, with anthropogenic nutrients from agriculture and waste hindering both coral resistance to bleaching and postbleaching recovery at cellular to ecological scales (*7*–*14*). Consequently, reducing anthropogenic nutrient enrichment to coral reefs has been a conservation priority (*15*, *16*). However, humans have altered nutrient regimes on coral reefs not only by increasing the flow of anthropogenic nutrients but also by disrupting the flow of natural nutrient subsidies provided by mobile animals. In contrast to human-caused nutrients, animal-derived nutrients deliver nitrogen and phosphorous in ratios that are beneficial to corals, potentially decreasing coral susceptibility to bleaching (*7*, *10*, *17*–*19*). Limited field studies have failed to conclusively demonstrate an effect of animal-derived nutrients on coral resistance to heat stress (*20*). However, recovery can be decoupled from resistance to bleaching, such that the amount of coral cover remaining after bleaching is a poor predictor of the degree and pace of coral recovery in subsequent years (*5*, *14*). Therefore, uncovering how natural nutrient subsidies influence the patterns and drivers of both resistance and recovery is essential to understand and predict coral reef resilience.

Seabirds are one group of mobile animals that play a key role in transferring and concentrating nutrients to nearshore coral reefs when they return to land from their oceanic foraging grounds (*21*–*23*). Seabird-derived nutrients, in turn, benefit coral reef fish growth, biomass, and ecosystem functioning (*21*, *24*, *25*). However, seabird populations are in rapid decline, with introduced mammalian predators, such as rats, on islands posing one of the greatest threats (*26*, *27*). In the tropics, non-native coconut palms replacing native breeding habitat cause additional disruptions to seabird-provided nutrient flows (*23*, *28*, *29*). Thus, one complementary solution to managing the flow of anthropogenic nutrients from land to coral reefs is eradicating invasive predators and restoring native vegetation to revive natural land-sea nutrient pathways provided by seabirds (*30*). Eradicating invasive mammals from islands is a key conservation strategy that is not only successful at restoring some seabird populations but also restoring the transfer of seabird-derived nutrients to islands and nearshore marine environments (*31*–*34*). Eradicating invasive mammals from islands has also recently been proposed as a powerful yet underused nature-based solution to promote climate change resilience (*35*). However, we lack sufficient knowledge of how seabird-derived nutrients interact with climate change–associated stressors to affect coral reef resilience. This information is essential to understand the projected impacts of island restoration initiatives for adjacent coral reefs and prioritize limited management resources.

Here, we test whether seabird-derived nutrients can enhance recovery of coral reefs following a major climate disturbance at scales ranging from individual coral colonies to islands. Coral recovery is primarily driven by growth of remnant colonies that survived the initial disturbance and by recruitment of new coral colonies (*36*, *37*). Limited evidence suggests that seabirds may aid both processes. For example, higher proportions of seabird-derived nutrients are found in corals near seabird colonies (*22*, *38*), and corals monitored for a single year grew faster near a seabird-rich island (*38*), suggesting a link between seabird nutrients and coral growth. However, we lack experimental evidence for how seabirds influence coral nutrient signatures and for how seabirds influence coral nutrients and growth over multiple years and following climate disturbances. Similarly, it is unclear how seabird-derived nutrients affect coral recruitment. However, there is evidence for higher crustose coralline algae (CCA) cover and herbivore biomass around seabird-rich islands following a bleaching event (*20*), both of which may have positive effects on coral recruitment and juvenile survival (*39*, *40*). Hence, we tested three main hypotheses related to coral reef recovery and its underlying mechanisms: (i) Seabirds increase coral growth rates by providing nutrients that are assimilated by corals. (ii) Seabirds enhance coral recruitment following a major marine heatwave and subsequent mass coral bleaching event. (iii) Seabirds speed recovery of coral cover and a return to predisturbance benthic community structure following a major marine heatwave and subsequent mass coral bleaching event.

To address these hypotheses, we harnessed a unique opportunity whereby islands with healthy seabird populations are interspersed with nearby islands with few seabirds in a remote atoll system in the Indian Ocean (*21*). The patchy distribution of seabirds [composed primarily of sooty terns (*Onychoprion fuscatus*), lesser noddies (*Anous tenuirostris*), and red-footed boobies (*Sula sula*)] on nearby islands is largely driven by the presence or absence of introduced rats, with introduced coconut palms on some islands contributing to further seabird reductions (*21*, *23*, *41*, *42*). As a result, seabird density, biomass, and nitrogen inputs are all orders of magnitude higher on rat-free islands (hereafter, “seabird islands”) compared to rat-infested islands (hereafter, “rat islands”) (mean seabird density, biomass, and nitrogen inputs on seabird versus rat islands = 1243 versus 1.6 seabirds/ha, 367 versus 0.7 kg/ha, and 190 versus 0.8 kg/ha per year, respectively) (*21*, *23*). The reefs in the study region are exposed to few other local anthropogenic stressors (*43*) but suffered extensive coral bleaching and mortality following Indo-Pacific wide marine heatwaves in 2015–2016 (*20*, *44*, *45*). From 2018 to 2021, we conducted a multiyear study of stable isotope values and growth rates of *Acropora* corals, using both a reciprocal transplant experiment and unmanipulated coral colonies. We focused on *Acropora* because it is the dominant coral at these sites and plays a key role in the resilience and functioning of reefs more generally (*46*–*48*). We also surveyed entire coral and benthic communities spanning from 1 year before the bleaching event to 6 years after bleaching and modeled *Acropora* recovery dynamics for the years between surveys. This combination of experimental and observational approaches allowed us to both establish causal pathways linking seabirds to mechanisms of reef resilience and document how seabirds influence recovery patterns in a natural system. In addition, our long-term experiments around rat-free and rat-infested islands enable predictions regarding the effects of invasive rat eradication and seabird restoration on coral recovery and the time scales over which these benefits may occur.

## RESULTS

### Corals assimilated seabird-derived nutrients

**Fig. 1.**
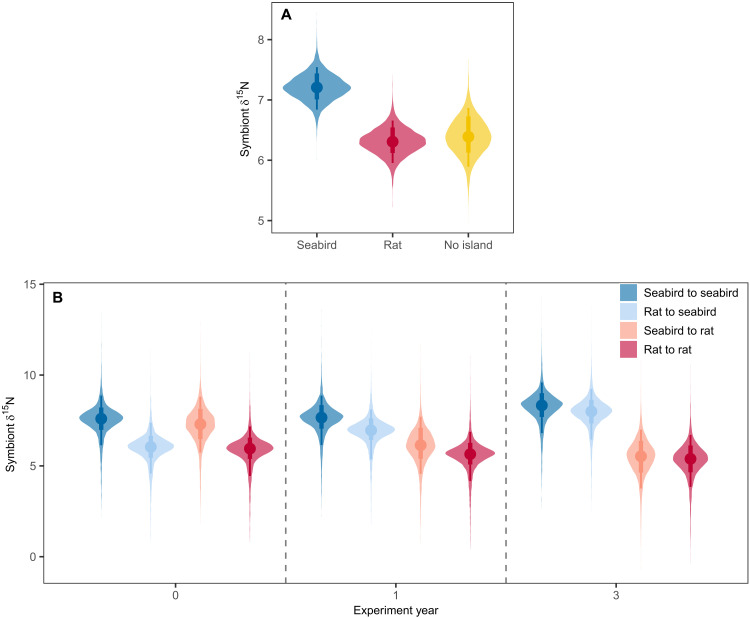
Effect of seabird versus rat presence on seabird-derived nutrients in coral symbionts, measured as δ^15^N. Posterior predictive distributions for natural *Acropora* colonies from (**A**) seabird (rat-free) islands, rat-infested islands, and control reefs with no nearby islands and (**B**) for *Acropora* colonies reciprocally transplanted between pairs of seabird and rat islands. Points represent median estimates, and lines represent 90 and 70% highest posterior density intervals (HPDIs).

We first established whether corals assimilate seabird-derived nutrients by comparing nitrogen stable isotope values (reported as δ^15^N, which is the ratio of isotopic nitrogen ^15^N to ^14^N relative to the ratio in standard reference atmospheric nitrogen) from *Acropora* corals collected within atoll lagoons near seabird islands, rat islands, and control reefs with no nearby islands (“no islands”). δ^15^N values provide a reliable tracer of seabird-derived nutrients, with values in coral symbionts accurately reflecting the nutrients available to and used by the coral holobiont, especially for highly autotrophic corals such as *Acropora* (*22*, *38*, *49*). Corals near seabird islands assimilated nutrients that were transferred by seabirds from their oceanic feeding grounds to these nearshore reefs. Coral colonies near seabird islands had ~1.1 times higher δ^15^N values in their symbionts, which is indicative of higher seabird-derived nutrients, than colonies from reefs near rat islands or no islands [estimated absolute difference = 0.9 and 0.8, respectively; 95% highest posterior density interval (HPDI) = 0.3 to 1.5 and 0.1 to 1.6, respectively] (Fig. 1A). By contrast, corals near rat islands had similar symbiont δ^15^N values than those near no islands (estimated absolute difference = 0.1, 95% HPDI = −0.7 to 0.8) (Fig. 1A).

Evidence from a reciprocal transplant experiment, which involved moving *Acropora* corals between pairs of seabird and rat islands, confirmed that the presence of seabirds caused the enriched nutrient values. At the beginning of the experiment, coral symbiont δ^15^N values only matched origin treatment, with corals from seabird islands having an estimated 1.2 times higher δ^15^N than those from rat islands (estimated absolute difference by origin treatment = 1.5, 95% HPDI = 0.6 to 2.3; estimated absolute difference by transplant treatment = 0.2, 95% HPDI = −0.5 to 1.0). However, the driver of nutrient signatures clearly shifted from origin treatment to transplant treatment throughout the course of the 3-year experiment, with the change beginning in year 1 (Fig. 1B). Corals that originated from rat islands and were transplanted to seabird islands exhibited a gradual increase in symbiont δ^15^N, with median values of 6.0 in 2018, 7.0 in 2019, and 8.0 in 2021. Conversely, corals that originated from seabird islands and were transplanted to rat islands exhibited the opposite pattern—dropping in δ^15^N values from 7.3 to 6.1 to 5.5. Meanwhile, corals that originated and remained at seabird islands maintained consistently high δ^15^N values (≥7.6), while those that originated and remained at rat islands had low δ^15^N values throughout (≤6.0). By the end of the 3-year experiment, symbiont δ^15^N values only matched transplant, but not origin, treatment; corals transplanted to seabird islands had 1.5 times higher δ^15^N than those transplanted to rat islands (estimated absolute difference by transplant treatment = 2.7, 95% HPDI = 1.8 to 3.6; estimated absolute difference by origin treatment = 0.4, 95% HPDI = −0.8 to 1.2) (Fig. 1B).

### Seabird-derived nutrients enhanced coral growth

**Fig. 2.**
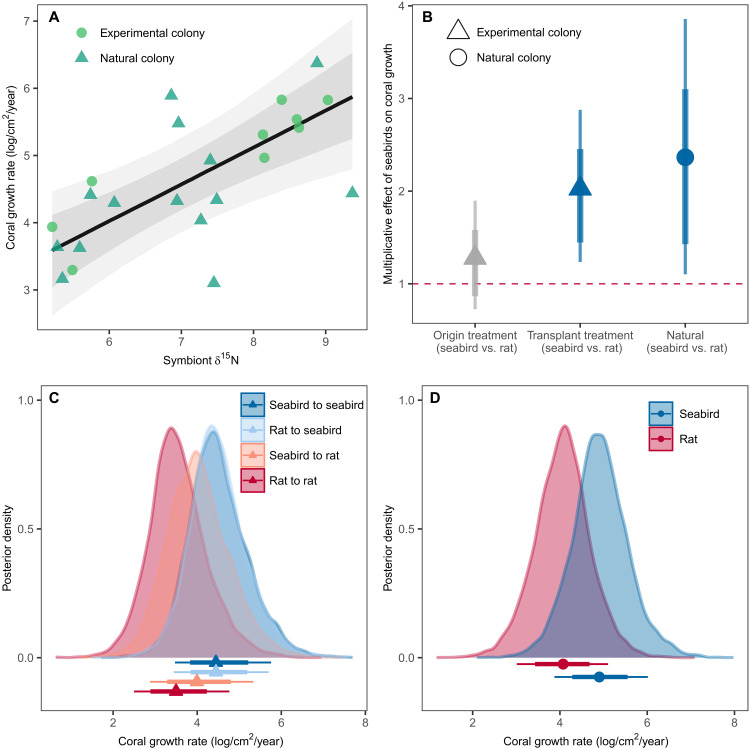
Effect of seabird-derived nutrients and seabird versus rat presence on coral growth rates. Posterior predictions presented for both experimental colonies (corals used in a reciprocal transplant experiment between pairs of seabird and rat islands) and natural colonies (unmanipulated corals from either seabird or rat island). (**A**) The effect of δ^15^N (a proxy for seabird-derived nutrients) in individual *Acropora* colonies on individual growth rates. Points are raw data, line represents conditional effect after controlling for colony size, and gray shading indicates 90 and 70% highest posterior density intervals (HPDIs). (**B**) Conditional effect of seabird presence on coral growth at an island level, with points above the dashed line indicating a positive effect of seabirds on growth. For experimental corals, “origin treatment” indicates whether corals started on a seabird or rat island, and “transplant treatment” indicates whether they were moved to a seabird or rat island for the duration of the experiment. Points represent median estimates, and lines represent 90 and 70% HPDIs. (**C** and **D**) Posterior predictive distributions for coral growth rates by treatment for (C) experimental colonies and (D) natural colonies. Points represent median estimates, and lines represent 90 and 70% HPDIs. All predictions are for a colony of average size.

Seabird-derived nutrients assimilated by corals boosted coral growth rates at both individual and island scales. We measured growth rates and sampled stable isotopes of both “experimental corals” (used in the reciprocal transplant experiment) and “natural corals” (tagged on nearby substrate but not manipulated or handled in any way). By pairing growth and isotope samples from the same *Acropora* colonies, we demonstrate that individual growth rates increased with increasing symbiont δ^15^N values (Fig. 2A). For each one-unit increase in symbiont δ^15^N, coral growth more than doubled (estimate = 2.1, 95% HPDI = 1.1 to 3.9).

At an island level, experimental coral colonies that were transplanted to seabird islands grew 2.0 times faster than those transplanted to rat islands (95% HPDI averaged over origin treatment = 1.2 to 3.2) (Fig. 2, B and C, and fig. S1). Regardless of origin, median growth rate per year for corals transplanted to seabird islands was approximately 85 cm^2^/year (for coral of average size; median for corals from seabird islands = 85.2, median for corals from rat islands = 85.4). Similar to patterns of δ^15^N in experimental corals, origin treatment did not have an overall effect on growth rate (median times difference in growth of corals from a seabird island compared to from a rat island = 1.3, 95% HPDI = 0.7 to 2.1). However, there was some evidence of an interactive effect between origin and transplant treatment, whereby corals transplanted to rat islands from seabird islands grew marginally faster than those from rat islands (median times difference in growth = 1.6, 95% HPDI = 0.6 to 3.2; median growth rate for coral of average size = 54.2 versus 33.0 cm^2^/year for seabird to rat versus rat to rat). Similar to the experimental corals, natural coral colonies also grew faster near seabird islands, with an estimated 2.4 times faster growth around seabird islands compared to rat islands (95% HPDI = 1.1 to 4.5) (Fig. 2, B and D).

### Coral recruitment was limited regardless of seabird presence

**Fig. 3.**
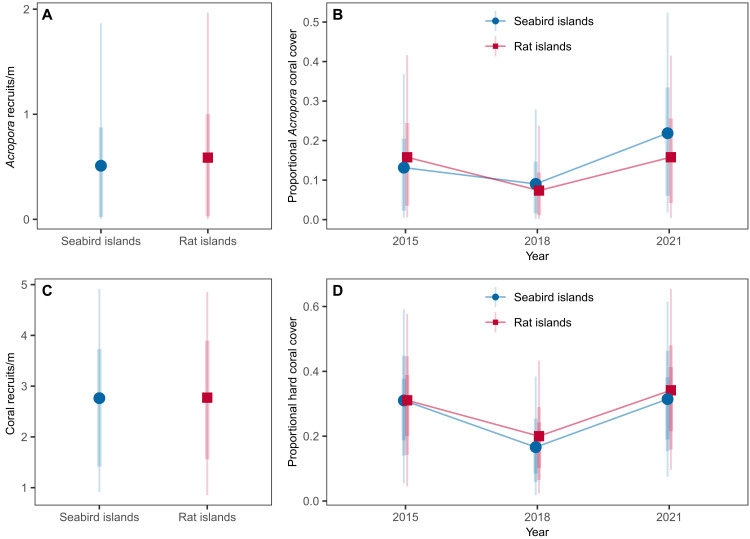
Reef-scale recruitment and coral cover around rat-free islands with abundant seabirds (seabird islands) versus rat-infested islands with few seabirds (rat islands). Posterior predictions for (**A** and **B**) *Acropora* corals only and (**C** and **D**) all genera combined. Recruitment was quantified in 2018 (3 years after bleaching), while coral cover was quantified in 2015 (before bleaching), 2018 (3 years after bleaching), and 2021 (6 years after bleaching). Points represent median estimates and lines represent 90 and 70% highest posterior density intervals (HPDIs).

Surveys conducted 3 years following a mass coral bleaching event found low juvenile coral density across all islands, regardless of rat status. At half of the islands (three seabird and two rat), we found zero *Acropora* recruits, and, overall, *Acropora* recruitment was estimated to be 0.51 m^−2^ around seabird islands versus 0.59 m^−2^ around rat islands (95% HPDI = 0.0 to 2.7 and 0.0 to 2.8, respectively; estimated difference, 95% HPDI = −0.1, −2.1 to 2.1) (Fig. 3A). Likewise, estimated recruitment of all corals was 2.8 recruits/m^2^ to both seabird and rat islands (95% HPDI = 0.6 to 5.6 and 0.5 to 5.7, respectively; estimated difference, 95% HPDI = −0.0, −2.0 to 2.0) (Fig. 3B). *Stylophora*, *Acropora*, and *Porites* comprised the majority of total recruits. While low numbers of *Acropora* and *Porites* recruits were spread throughout the study region, *Stylophora* recruits were only present at three islands, with the highest densities on two islands in relatively close proximity to each other (within the southwest corner of Peros Banhos atoll).

Similarly, both *Acropora* and overall coral size distributions were similar between seabird and rat islands 6 years after bleaching, not only in median size but also in skewness and SD (estimated difference, 95% HPDI for *Acropora*: median = 0.1, −0.3 to 0.4; skewness (α) = −1.2, −5.1 to 2.5; SD (σ) = 0.0, −0.3 to 0.2. All corals: median = 0.0, −0.4 to 0.5; α = −0.6, −2.6 to 1.6; σ = 0.0, −0.2 to 0.2) (fig. S2). Furthermore, there was no evidence for a spike in recruitment or conversely for a shift toward larger colonies, as evidenced by all size distributions following the predicted lognormal distributions with a skewness parameter of approximately 0 (estimated α, 95% HPDI for *Acropora* seabird = −0.1, −2.9 to 3.5; rat = −1.2, −4.6 to 2.3; for all corals seabird = 0.7, −1.4 to 2.8; rat = 0.1, −2.0 to 2.1).

### Corals recovered rapidly, with faster *Acropora* recovery near seabird islands

Given faster coral growth around seabird islands, combined with low overall recruit densities, we expected hard coral cover to rebound more quickly around seabird islands due to faster growing remnant colonies. We observed similar initial declines in hard coral, followed by rapid coral recovery regardless of rat status, as determined by repeated benthic surveys in 2015 (before bleaching), 2018 (~3 years after bleaching), and 2021 (~6 years after bleaching) (Fig. 3D). Hard coral cover dropped from approximately 31% of the benthos in 2015 to 18% in 2018 (estimated difference, 95% HPDI in 2015 versus 2018 cover seabird = 13.5, 2.9 to 24.7; rat = 10.0, 1.4 to 19.6). Relative hard coral then increased by 70 to 90% between 2018 and 2021, with absolute increases of 13.1% around rat islands and 13.9% around seabird islands between 2018 and 2021 (95% HPDI = 3.0 to 23.3 and 3.0 to 25.1). By 2021, median hard coral cover at all islands was equivalent to prebleaching cover (estimated difference, 95% HPDI in 2021 versus 2015 cover seabird = 0.3, −6.8 to 8.0; rat = 2.6, 4.2 to 10.6).

**Fig. 4.**
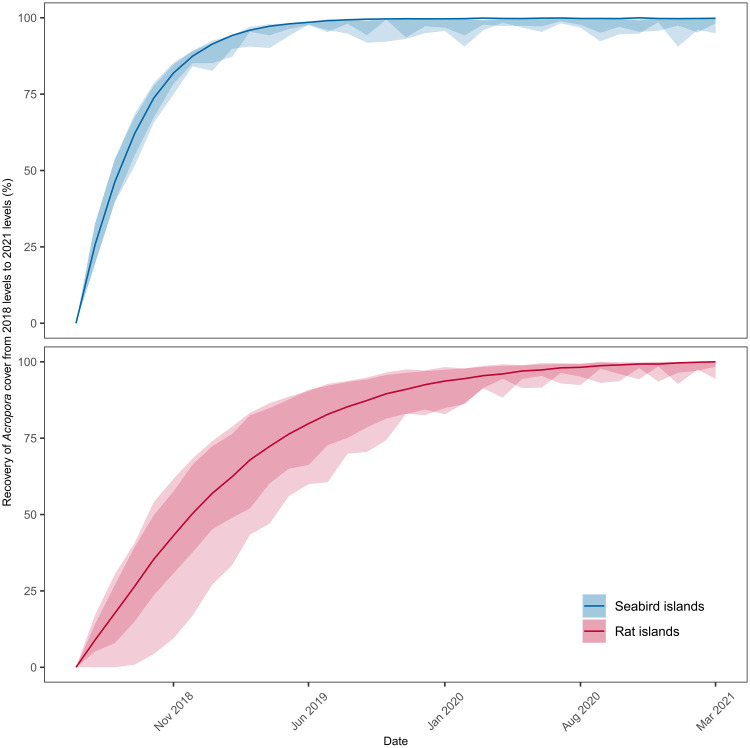
Reconstructed recovery trajectories of *Acropora* coral cover around rat-free islands with abundant seabirds (seabird islands) versus rat-infested islands with few seabirds (rat islands). Recovery of *Acropora* cover shown between April 2018 (3 years after bleaching) and May 2021 (6 years after bleaching). Lines represent median estimates, and shaded areas represent 70 and 90% confidence intervals.

Branching *Acropora* remained the dominant genus in all years, and temporal trends in *Acropora* cover were qualitatively similar to those for overall hard coral cover, with one exception. While absolute *Acropora* cover around rat islands recovered to prebleaching levels (estimate = 0.1, 95% HPDI = −7.0 to 6.6), around seabird islands *Acropora* not only recovered but also was 7.7% higher than prebleaching levels (95% HPDI = 0.1 to 18.6) (Fig. 3B). Modeling of *Acropora* cover between 2018 and 2021, parameterized with data on growth rates and recruitment from the study, suggests that *Acropora* recovered more quickly around seabird islands. Recovery of median *Acropora* cover to 90% levels took 8 months at seabird islands (95% confidence interval = 8 to 12), whereas at rat islands this proportion of recovery took 18 months (95% confidence interval = 13 to 23) (Fig. 4). Thus, total recovery time was approximately 3 years and 8 months (3.67 years) around seabird islands versus 4 years and 6 months (4.50 years) around rat islands.

In addition to coral cover, coral communities changed around both seabird and rat islands 3 years after bleaching (fig. S3). Following bleaching, the cover of *Acropora*, branching Pocilloporidae (e.g., *Pocillopora* and *Stylophora*) and *Isopora* corals all decreased, while cover of some massive corals, such as *Porites, Platygyra*, and corals formerly classified as Faviidae (now mostly Merulinade, e.g., *Dipsastraea*, *Echinopora*, *Favites*, and *Goniastrea*) remained relatively stable. However, as for coral cover, coral communities recovered rapidly, returning to their original structure within 6 years of the bleaching event (fig. S3).

### Benthic recovery trajectories were more dynamic near seabird islands

**Fig. 5.**
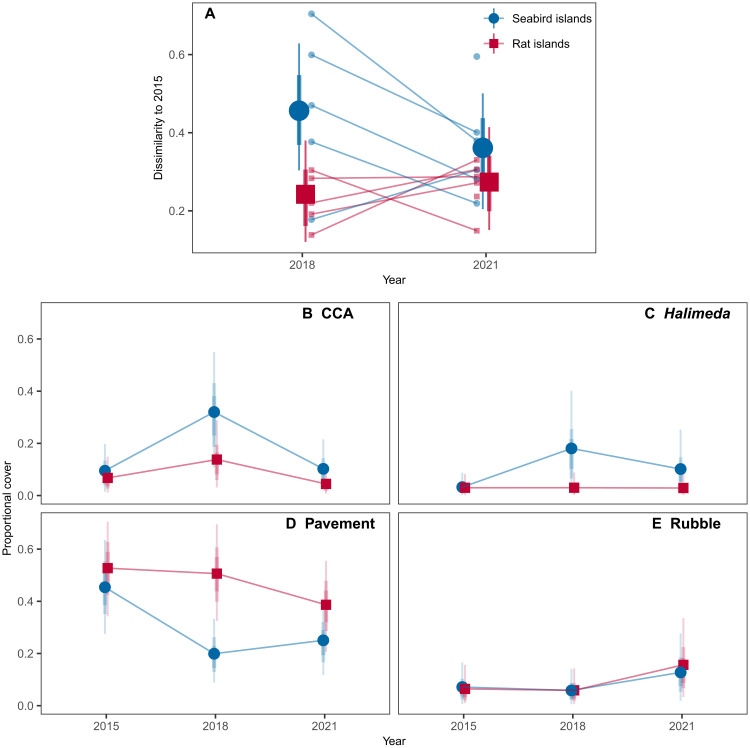
Recovery of all benthic groups, based on repeated surveys around rat-free islands with abundant seabirds (seabird islands) versus rat-infested islands with few seabirds (rat islands) in 2015 (before bleaching), 2018 (3 years after bleaching), and 2021 (6 years after bleaching). (**A**) Posterior predictions for reef-scale benthic recovery, measured as Bray-Curtis dissimilarity to prebleaching baseline of the whole benthic community. Small points and connecting lines represent individual islands through time. Large points and lines represent median estimates and 90 and 70% highest posterior density intervals (HPDIs). (**B** to **E**) Posterior predictions for proportional cover of broad benthic groups, except hard coral cover (see Fig. 3D). Points represent median estimates, and lines represent 90 and 70% HPDIs.

Expanding beyond hard coral to overall benthic communities, rat status influenced community structure and trajectories through time (Fig. 5A and fig. S3). Specifically, although benthic communities 3 years after bleaching around seabird islands were more different from their prebleaching states compared to those around rat islands (estimated difference, 95% HPDI in Bray-Curtis dissimilarity in 2018 = 0.21, 0.04 to 0.38), by 6 years after bleaching, seabird benthic communities were exhibiting greater return toward their initial state (estimated difference, 95% HPDI = −0.09, −0.25 to 0.07). By contrast, distance from prebleaching state around rat islands in 2021 compared to 2018 was either steady or increasing (estimated difference, 95% HPDI = 0.03, −0.11 to 0.17).

The rapid change and return in benthic community structure around seabird islands was primarily driven by temporary increases in calcifying algae (CCA and *Halimeda*). Around seabird islands, absolute percent cover of CCA and *Halimeda* in 2018 was 22.4 and 14.8% higher than in 2015 (95% HPDI = 10.3 to 35.2 and 2.7 to 30.8, respectively) but, by 2021, was only 0.6 and 6.8% higher, respectively (95% HPDI = −2.9 to 4.4 and 1.2 to 16.8) (Fig. 5, B and C). This increase in calcifying algae around seabird islands was accompanied by a decrease in pavement (hard substrate, including turf algae), whereas around rat islands, pavement continued to dominate the benthos across all years (median cover in 2015, 2018, and 2021: seabird = 45.4, 20.0, and 25.0%; rat = 52.7, 50.6, and 38.7%, respectively) (Fig. 5D). Last, there was an increase in rubble 6 years after bleaching around both seabird and rat islands compared to prebleaching reefs (difference, 95% HPDI in 2021 versus 2015 cover seabird = 5.4, 0.2 to 14.0; rat = 9.0, 1.1 to 21.4) (Fig. 5E).

## DISCUSSION

Identifying local solutions that bolster coral reef resilience to climate change is critical for the persistence of these important, yet vulnerable, ecosystems. Here, we show that seabird-derived nutrient inputs drive faster coral growth rates at both colony and island scales. There were also faster recovery rates in the percent cover of *Acropora* and more dynamic benthic recovery patterns around rat-free islands with abundant seabirds. Combined, these findings suggest that eradicating rats and restoring seabird populations could play a role in bolstering rapid coral reef recovery following climate disturbances. At the same time, the rapid return of coral cover and community structure around all islands suggests that multiple processes contribute to high overall resilience in this system.

That nesting seabirds provide cross-ecosystem nutrient subsidies to tropical coral reefs is now well established (*21*–*23*, *33*, *38*). Similar to the patterns observed here, ~1 to 2 times higher δ^15^N values have likewise been found in coral symbionts and host tissue near islands with abundant seabird populations, with elevated seawater nitrate concentrations (~15 to 50 times) close to the same islands, relative to sites farther offshore (*22*, *38*). However, the observed shift in δ^15^N signatures in transplanted coral colonies provides experimental field evidence that coral reef organisms quickly begin assimilating seabird-derived nutrients when given the opportunity, but it takes approximately 3 years for corals to fully acclimate to local nutrient regimes. Not only do corals assimilate seabird-derived nutrients but these nutrients, in turn, boost coral productivity measured as growth. Although faster growth of both corals and herbivorous fishes around islands with abundant seabird populations has previously been demonstrated (*21*, *25*, *38*), this study matches individual growth rates with individual isotopic signatures of coral reef organisms. In doing so, we demonstrate that even within islands, individuals that assimilated more seabird-derived nutrients grow faster. The causal link from seabird presence to enhanced nutrients to enhanced growth rates established by our reciprocal transplant experiment provides convincing evidence that the faster growth rates are caused directly by the assimilation of seabird-derived nutrients within individual colonies. This distinction between direct and indirect effects of seabird nutrients on growth is important given that other factors that influence growth rates, such as predator biomass and territorial behavior of fish, can also differ between rat-free and rat-infested islands (*21*, *50*).

In addition to coral growth rates, recruitment is the other major driving force behind recovery of coral cover following disturbance events. However, the relative importance of these two mechanisms can vary (*37*). The low juvenile coral densities across all islands suggest that initial recovery on these reefs was primarily driven by growth of remnant colonies, rather than recruitment, similar to some other isolated reef systems (*51*, *52*). Recruitment is temporally variable and reduced densities of coral recruits, and juveniles within several years of major coral bleaching events have similarly been documented in several other Indian Ocean reef systems, including in the Chagos Archipelago in 2017 (*45*, *51*, *53*–*55*). Given that percent coral cover is one driving factor of recruitment rates (*51*) and coral cover recovered rapidly on our reefs, we could reasonably expect a similar increase in coral recruits within that time frame. However, coral size distributions measured 6 years following the bleaching event, after full recovery of coral cover and coral community structure, still showed no evidence of a recruitment pulse. The lack of recruitment was likely because we focused on shallow lagoonal reefs, which often have lower recruit densities than forereefs and many deeper lagoonal reefs (*45*, *56*, *57*). The low recruit densities, along with similar coral cover between seabird and rat islands, may also partially explain why there was no detectable effect of seabird versus rat presence on recruitment.

A balance of other factors relating to larval supply, available settlement habitat, and postrecruitment processes may further explain the comparable number of recruits between seabird and rat islands, despite a strong effect of seabird presence on coral nutrients and growth. For example, anthropogenic nutrient enrichment decreases multiple stages of successful coral reproduction and recruitment (*12*), but it is unknown how seabird-derived nutrients influence coral reproduction. Seabird nutrients do, however, alter trade-offs between growth and reproduction in parrotfish (*25*). Available settlement habitat can also influence coral recruitment, and we hypothesized that increased CCA around seabird islands would provide preferred substrate (*20*, *39*). However, our results suggest that settlement substrate was not a limiting factor, especially given the low densities of juvenile corals. Last, postrecruitment processes are also crucial factors regulating the presence of recruit and juvenile corals on reefs. For example, herbivorous fishes, which have higher biomass around seabird islands (*20*, *21*), can benefit juvenile corals by preventing macroalgal overgrowth (*40*), but parrotfish can also induce mortality via incidental predation (*58*). Ultimately, that there was no difference in coral recruits between seabird and rat islands is the result of many different driving forces, with further research need to disentangle the influence of seabird-derived nutrients on these multiple processes.

Scaling up from the mechanisms behind recovery to reef-wide recovery dynamics, the rapid recovery of coral cover is promising. That we observed recovery of hard coral cover and coral communities within 3 to 6 years is faster than expected on the basis of the 1998 mass bleaching event, during which full recovery took ~10 years in the Chagos Archipelago and ~15 years in the wider region, when it occurred at all (*45*, *59*, *60*). Emerging evidence from forereefs within the Chagos Archipelago (*61*) and lagoonal and shallow reefs elsewhere in the Indian (*62*) and Pacific (*63*) Oceans similarly shows recovery of coral cover to prebleaching levels within 3 to 6 years of the 2015–2016 mass bleaching event at some sites. Notably, these reefs are all protected from direct, local human impacts and thus suggest that protected and remote areas play a role in enhancing reef resilience to bleaching events (*64*). Beyond aggregate measures of hard coral cover, trajectories of dominant coral taxa and coral community structure are key metrics of reef recovery, as these characteristics can influence the ecosystem functions and services provided by corals (*65*, *66*). In contrast to some other locations where coral communities shift following bleaching events (*52*, *67*), we found that although coral community structure initially changed 3 years after bleaching, it had returned to its original state within 6 years. This rapid community recovery is likely partially due to initial community structure, as *Acropora*-dominated reefs tend to exhibit quicker return to prebleaching communities (*67*). That coral cover remained above 15% even after bleaching, with growth of remnant colonies rather than recruitment driving recovery, likely further facilitated the return to initial community structure. Combined with the rapid return of hard coral cover, the simultaneous recovery of coral communities indicates that these reefs and the functions they provide are highly resilient, at least to bleaching events of this magnitude.

This study provides evidence that seabirds are an important component of coral reef resilience for several reasons. First, our modeling of *Acropora* cover during the interval between 3 and 6 years after bleaching predicts that *Acropora* recovered faster around islands with seabirds. This interval may be a critical threshold, as the median time between successive bleaching events was 5.9 years in 2016, a reduction from 27 years in the 1980s and with increased frequency of bleaching expected (*3*). Thus, even small reductions in recovery times during this window may be key to maintaining coral cover and associated functions. Given that *Acropora* was the dominant genus, it is likely that overall hard coral cover followed the same pattern of quicker recovery around seabird islands. However, *Acropora* corals may be differentially affected by seabird nutrients, as they are fast-growers and exhibit different responses to elevated and skewed nutrient conditions than other coral genera (*19*). Here, *Acropora* cover rebounded to higher than prebleaching levels around seabird islands, suggesting that seabird nutrients may have given them an additional advantage over other corals during the recovery phase. By contrast, it is possible that around rat-infested islands other coral genera grow faster, either due to the absence of nutrient subsidies or due to less competition with fast-growing *Acropora*. Even if this is the case, the population dynamics of branching *Acropora* can be particularly important to the resilience and functioning of reefs, as it is often the main driver of both coral loss and recovery from bleaching events (*46*) and is considered a keystone structure due to its provision of complex habitat for fish (*47*, *48*). Furthermore, *Acropora* contributes disproportionately to essential services, including wave dissipation and reef growth, which are particularly important for protecting atoll islands at risk of increased coastal erosion and submergence with sea level rise (*61*, *68*). Comparing the benefits of seabird-derived nutrients for corals from a range of genera and growth strategies and how this, in turn, influences competition and other dynamics on reefs dominated by different coral genera will provide additional insights for how seabirds may shape coral communities into the future. Combining these ecological studies with genetic work, for example, to determine whether symbiont identity differs as a function of seabird status, will further our understanding of the mechanisms underlying the effects of seabird-derived nutrients on corals.

In addition to live hard coral, other benthic organisms are also important components of reef ecosystems and, therefore, of reef functioning and recovery. For example, CCA binds reefs and contributes to carbonate accretion (*69*), and rubble habitats harbor high invertebrate diversity (*70*). Overall, islands with seabirds were more dynamic and underwent more rapid benthic changes than rat-infested islands, which could be important to the long-term maintenance of both coral cover and other benthic groups that support ecological functions. For example, seabird islands had large booms and then busts of calcifying algae following the bleaching event. A similar rapid shift to CCA following a bleaching event may have helped stabilize coral cover in Palmyra Atoll, where rats were eradicated 4 years earlier, but whether these patterns relate to seabirds remains untested (*71*). By contrast, on rat islands, the dominant cover after bleaching was pavement (a category including turf algae). Although a shift to pavement and highly productive short algal turfs following disturbance may be part of typical successional patterns, it can also facilitate transitions to longer sediment-laden turf or fleshy macroalgae, which, in turn, can inhibit recovery of corals and ecosystem functions (*72*–*75*). Moreover, emerging evidence suggests that on Indo-Pacific reefs, low-lying algae is the major group replacing hard coral cover (*76*), and a switch from coral to turf algae is the second most common phase shift on tropical reefs worldwide (*77*). Thus, high cover of pavement and turf algae, combined with more static benthic trajectories, around rat-infested islands means that these benthic communities may be increasingly likely to get locked into alternative states following bleaching. While seabird islands were rebounding toward their initial state 6 years following the bleaching event compared to 3 years after bleaching, community dissimilarity from the baseline was stable or increasing around rat-infested islands.

Combined, these findings provide key insights into the use of island restoration, including eradicating rats and other invasive predators, as a conservation strategy to boost coral reef resilience. First, there is a high likelihood that restoring healthy populations of breeding seabirds would result in increased assimilation of seabird-derived nutrients by corals within 1 year and full assimilation within 3 years. The restoration of seabird populations and their associated nutrient flows to coral reefs can occur relatively rapidly (within several decades) of eradicating rats from islands (*33*), making this a viable short-term action. However, this timeline is for remote islands in the Indian Ocean, where there are few or no other invasive predatory mammals, native vegetation is still present, and nearby healthy populations of seabirds can act as sources (*33*). In locations where these conditions are not met, additional management actions will likely be necessary in conjunction with rat eradication to successfully restore seabird populations, including eradicating other predatory invasive mammals (e.g., cats), removing abandoned coconut plantations, replanting preferred native vegetation, social attraction, or translocation of seabirds, and protecting seabirds from direct exploitation (*26*, *42*). Once seabird-derived nutrient flows are restored, they would likely result in increased coral growth rates over the same time frame of 1 to 3 years. Likewise, another study investigating the effects of seabird-derived nutrients on coral growth rates also saw faster growth in a 1-year transplant experiment (*38*). That we observed increased growth throughout the 3-year experiment, combined with enhanced growth in natural coral colonies and a direct link to higher seabird nutrients in individual colonies, suggests that this will not be a temporary boost to coral growth but instead will be maintained in the long-term. By contrast, rat eradication and other techniques to restore seabird populations may not directly benefit recruitment and/or survival of juvenile corals, especially in a system and habitat (i.e., lagoonal reefs) with low overall recruitment. Similarly, the overall rapid coral recovery on isolated reefs suggests other protections (e.g., protected areas and reduced human-caused nutrient pollution) may foster the return of coral cover, but seabird restoration would likely further speed coral recovery on *Acropora*-dominated reefs to <4 years. Ultimately, given the potential for seabirds to benefit coral resilience to climate change, combined with their documented benefits for coral reef ecosystem functioning (*21*, *24*, *33*), restoring seabird populations via strategies including rat eradication should be prioritized alongside other local protections for coral reefs, especially when combined with continued progress toward reducing global emissions.

## MATERIALS AND METHODS

### Study system

We conducted this study in the northern atolls of the Chagos Archipelago, Indian Ocean (5°50′ S, 72°00′ E). The entire region is protected as part of a very large (640,000 km^2^) Marine Protected Area, and all islands in the northern atolls have been uninhabited by humans since the 1970s (*43*). Because of its remote location and protection from local human impacts, the region is often considered a “baseline” for the Indian Ocean (*43*).

The Chagos Archipelago supports 18 species and >280,000 pairs of breeding seabirds, with several species breeding in regionally and globally important numbers (*41*, *43*). Sooty tern, lesser noddy, and red-footed booby make up 96% (70, 18, and 8%, respectively) of the archipelago’s seabird assemblage (*41*). However, rats, which cause substantial reductions in certain seabird populations via direct predation (*27*), were introduced to some islands hundreds of years ago (*41*). Non-native coconut palms, which replace native vegetation and limit availability of seabird breeding habitat, were also introduced to some islands around the same time (*42*, *43*).

For this study, we focused on 12 islands spread across three atolls for which the effect of rats and non-native vegetation on seabirds, seabird-derived nutrient flows, and coral reef ecology have been well studied (*21*, *23*). Six islands were rat-infested, and six were rat-free, with percent cover of non-native coconut palms ranging from 0 to 66% on rat-free islands and 55 to 91% on rat-infested islands (*23*). Both rats and coconut palms reduce seabird biomass and diversity on these islands, but rats have a stronger negative effect on biomass, and coconut palms have a stronger negative effect on diversity (*23*). After accounting for any effect of non-native vegetation, total seabird biomass is estimated to be >160 times higher on rat-free than rat-infested islands (*23*). Similar to the archipelago as a whole, terns, noddies, and boobies were present on all rat-free islands and comprised the majority of biomass, with frigatebirds, shearwaters, and tropicbirds also present on some rat-free islands (*21*, *33*). By contrast, biomass of all families was lower on rat-infested islands, with frigatebirds and shearwaters completely absent (*21*, *33*). As a result, rat-free islands are characterized by approximately 250 times higher inputs of seabird-derived nutrients than rat-infested islands, which subsequently flow to nearshore coral reefs where they are available to coral reef organisms (*21*, *23*). Therefore, we refer to the rat-free islands with abundant seabird populations and high inputs of seabird-derived nutrients as "seabird islands," and the rat-infested islands with few seabirds and limited seabird-derived nutrient inputs as “rat islands.”

We focused our study on shallow reefs (<4 m) on the lagoonal sides of atoll islands within several hundred meters of shore. Previous work on these reefs has shown that seabird-derived nutrients are assimilated by algae, sponges, and herbivorous reef fish around seabird islands, resulting in altered behavior, faster growth, higher biomass, and enhanced ecosystem functioning of fish than around rat islands (*21*, *24*, *25*, *50*). In addition, sampling of terrestrial and marine organisms across the island-reef interface indicate that the proportion of seabird-derived nutrients declines with increasing distance from seabird islands, providing strong evidence that nutrients are being transported to islands by seabirds and then being discharged onto the reef (*21*, *23*, *33*). All study islands were at least 3 km apart, which is much greater than the documented spatial extent of seabird-derived nutrients within coral reef systems based on both seawater measurements of nitrates and isotopic values in coral reef organisms (typically <400 m, maximum of 1200 m) (*22*, *33*).

Coral reefs throughout the Indo-Pacific, including in the Chagos Archipelago, experienced an extreme marine heatwave in 2015–2016 that led to mass coral bleaching and mortality in 2016–2017 (*3*, *45*). Relative hard coral declined by 32% on our study reefs between 2015 and 2018, the magnitude of which was not affected by rat or seabird presence (*20*). Although benthic community structure was similar between reefs near seabird and rat islands before the bleaching event, by 2018, calcareous algae (*Halimeda* and CCA) had become dominant on seabird, but not rat, islands (*20*).

### Hypothesis 1: Seabirds increase coral growth rates by providing nutrients that are assimilated by corals

To determine the influence of rat presence on uptake of seabird nutrients and growth of corals, we used both natural coral colonies and experimentally transplanted coral colonies. We focused on branching *Acropora*, as it is the most abundant coral at these sites. In addition, a reduction in *Acropora* was a primary driver in the loss of hard coral cover between 2015 and 2018, during which time absolute hard coral and *Acropora* declined by 10.6 and 7.8%, respectively (*20*).

#### Reciprocal transplant experiment

We conducted a reciprocal transplant experiment at two pairs of islands (one seabird and one rat island per pair), with one pair in each of two atolls (Salomon Atoll and the Great Chagos Bank) (fig. S4). From each island, five branching *Acropora* colonies of similar size and morphology were sampled, with three fragments approximately 5 cm in length taken from each colony. One fragment was wrapped in aluminum foil and frozen at −20°C for later analysis, one fragment was transplanted to the same island, and one fragment was transplanted to the paired island. This resulted in 10 coral fragments at each island—five that originated from the same island and five that originated from the paired island of opposite rat status. Coral fragments were transplanted as quickly as possible following collection and moved between islands in buckets of ambient seawater. Fragments were attached to dead reef structure using zipties and marked using numbered cattle tags. Initial setup occurred in May 2018, and corals were revisited in March 2019, March 2020, and April to May 2021. During each revisit, remaining coral colonies were remeasured (see below), and a small fragment was again taken and frozen for later analysis.

#### Natural coral colonies

To supplement nutrient sampling from the experimental coral colonies, we sampled branching *Acropora* colonies at an additional 11 sites in May 2018 and March 2019 (fig. S4). Samples were taken from four seabird islands and four rat islands across three atolls (Salomon, Great Chagos Bank, and Peros Banhos), as well as from three “control” sites in Peros Banhos (*n* = 4 to 5 colonies per site). The control sites were lagoonal reefs on raised knolls (3 to 6 m in depth) that were 2.3 to 3 km from the nearest island and used to account for any effects of nearby islands. As above, a ~5-cm fragment was sampled from each colony and immediately wrapped in aluminum foil and frozen at −20°C until laboratory analysis.

To further complement the reciprocal transplant experiment, we tracked growth of natural branching *Acropora* colonies. *Acropora* colonies of similar size and morphology were haphazardly selected, with at least 2 m between replicate colonies. We tracked individual colonies by attaching cattle tags to nearby substrate, with colonies at five islands tagged in 2018 and colonies at an additional seven islands tagged in 2019 (*n* = 5 to 7 colonies per island). We re-revisited sites annually from 2019 to 2021 during the same months as the reciprocal transplant experiment, but, in 2020, we only visited five islands due to a shortened expedition caused by the COVID-19 global pandemic. No tagged corals were found at three islands (South Brother, Nelson Island, and Grande Ile Mapou), leaving a total of nine islands (five seabirds and four rats) with corals that were both tagged and remeasured (fig. S4). During the final visit (2021), a small fragment from each colony was taken, wrapped in aluminum foil, and frozen at −20°C for later analysis to enable matching of individual growth rates from natural colonies to their individual nutrient signatures, as for the experimentally transplanted corals.

#### Coral nutrient analysis

To measure the assimilation of seabird-derived nutrients by corals, we used stable isotope analysis of δ^15^N. Seabird-derived nutrients are enriched in δ^15^N compared to other nitrogen sources; thus, higher δ^15^N values in coral reef organisms indicate greater assimilation of seabird-derived nutrients (*21*–*23*, *33*, *38*). Corals contain both animal host tissue and algal symbiont components. Here, we focus on δ^15^N of the symbiont component because *Acropora* corals rely on their symbionts for food and nutrients (*49*). Therefore, these values reflect the nutrients available to and used by corals, and symbiont and host δ^15^N values are nearly identical (*22*, *49*). Furthermore, δ^15^N values of symbionts have been used as indicators of assimilation of seabird-derived nutrients by corals in previous studies (*22*, *38*).

In the laboratory, coral tissue was removed from the skeleton using an airbrush with Milli-Q water and homogenized. Host and symbiont fragments were separated by centrifuging, with each fragment stored at −20°C until freeze drying them for ~24 to 72 hours and grinding them into a fine powder using a mortar and pestle. Isotopic analysis was conducted at the University of Southampton (UK) using an Elementar Vario PYRO Cube Elemental Analyzer interfaced with an Isoprime VisION continuous flow isotope ratio mass spectrometer. Coral host and symbiont samples were weighed out in clean tin capsules on a Sartorius ME5 microbalance and combusted at 1120° with the addition of pure oxygen. The resulting combustion gases of NOx were subsequently reduced to N_2_ in the reduction column that was held at 850°C. The elemental ratios were determined by the thermal conductivity detector (TCD) and isotope ratios by the isotope ratio mass spectrometer (IRMS). Sulfanilamide was used as an elemental standard for %N. USGS 40 and USGS 41 were used as international reference materials (U.S. Geological Survey, Reston, VA, USA) for the normalization of isotope ratios.

#### Coral growth measurements

For both the reciprocal transplant and natural coral colonies, we measured growth as change in planar area. We chose this standard growth metric because it is relevant for coral demography (*78*) and can be used as a proxy for three-dimensional measurements (*79*), while being minimally invasive and reducing time in the field compared to other methods (e.g., alzirin staining, individual branch extension, and three-dimensional photogrammetry) (*80*). At every visit, we photographed each coral colony from directly above using a Canon S110 camera, with a scale bar placed level with the upper surface of the colony. All images were analyzed using ImageJ/FIJI (*81*), with planar area measured by outlining the outer edge of the colony using the polygon tool. We calculated growth as the change in planar area (in square centimeters) between subsequent measurements and converted it to a rate by dividing by the number of days between measurements.

#### Statistical analyses

To determine the overall effect of rat presence/absence on coral nutrients, we first compared nutrient signatures of all corals collected in 2018 for use in the reciprocal transplant experiment, and all natural colonies collected in either 2018 or 2019. This resulted in samples from 15 sites, with only one sample per individual coral (*n* = 74 colonies). We used a multilevel Bayesian model with δ^15^N in coral symbionts as our response to indicate assimilation of seabird-derived nutrients and seabird versus rat status as our explanatory variable and to compare rat-free islands with abundant seabirds (seabird islands), rat-infested islands with few seabirds (rat islands), and control reefs with no nearby islands (no islands). We included island nested within atoll as group-level effects to account for the hierarchical nature of our sampling design and nonindependence among coral samples from the same location.

To further test the drivers of coral nutrients and the time scales over which their nutrient signatures can change, we focused on analysis of δ^15^N in the reciprocal transplant corals that were remeasured at least once (*n* = 18 colonies). Using another multilevel Bayesian model, we tested for an effect of origin treatment (seabird versus rat), transplant treatment (seabird versus rat), experiment year (2018 = year 0, 2019 = year 1, and 2021 = year 3), and all two-way interactions, while including individual colony nested within atoll as group-level effects.

To link δ^15^N values and growth rates within individual corals, we tested for an effect of symbiont δ^15^N measured in 2021 on growth rate of natural and experimental colonies in the previous year (*n* = 23 colonies). We used this final time point because we had isotope data for all remaining natural colonies, and any legacy effects of origin treatment on transplanted coral δ^15^N had disappeared by this time (see Results). We used log-transformed coral growth rate to improve model fit, included symbiont δ^15^N along with previous coral planar area and type of colony (natural or experimental) as predictor variables, and again used island nested within atoll as group-level effects.

Last, we tested the effects of treatment on coral growth in both natural and experimental colonies at island scales (*n* = 17 natural colonies and 18 experimental colonies). As above, we log-transformed coral growth rate and included previous planar area as an additional covariate in both models. For natural coral colonies, seabird versus rat status was our main explanatory variable with year and individual colony nested within island nested within atoll as group-level effects to account for both spatial and temporal nonindependence. For experimental colonies, origin treatment, transplant treatment, and their interaction were our main predictor variables, with individual colony nested within atoll and year as group-level effects.

All models were run with weakly informative priors for four chains, with 3000 iterations and a warm-up of 1000 iterations per chain, using the brms package in R and implemented in STAN (*82*, *83*). We assessed model convergence and fit using posterior predictive checks, traceplots, and the Gelman-Ruban convergence diagnostic (R-hat) (*84*).

### Hypothesis 2: Seabirds enhance coral recruitment following a major marine heatwave and subsequent mass coral bleaching event

#### Coral recruit and size distribution surveys

To test whether recruitment and/or survival of juvenile corals differed between seabird and rat islands, we conducted focused surveys for coral juveniles. The size classification of recruit and juvenile corals varies within the literature (*57*), but we surveyed corals with a maximum diameter of ≤5 cm for several reasons: (i) This size cutoff is commonly used (*54*), including at other locations in the Indian Ocean (*53*); (ii) 5 cm represents a transitional size, with corals of >5 cm experiencing much lower mortality rates than those of <5 cm and are likely to represent the next generation of adults (*56*, *85*); and (iii) corals of this size range are expected to have settled in the previous 1 to 2 years (*56*, *85*), which suggests our surveys conducted in May 2018 included any corals that settled following the 2015–2016 mass bleaching event. In doing so, we acknowledge that any observed differences could be due to either differential rates of settlement (i.e., supply) and/or differential survival of recruits. However, given that we are ultimately interested in how seabirds influence coral recruitment and how this, in turn, affects recovery, this distinction is not important to our aims. We counted, measured (diameter to the nearest 1 cm), and identified to genus all juvenile corals within 12 quadrats of 0.25 m^2^ (50 cm by 50 cm) at each of 10 islands (five seabird and five rat) (fig. S4). At each island, three quadrats were placed haphazardly along each of four 30-m transects used for benthic surveys (see below).

In 2021, we conducted additional surveys of coral size distribution because shifts in size distribution could indicate differential recruitment and juvenile survival and are also relevant to coral demography and functional role (*86*, *87*). We conducted 6 to 13 transects of 10 m within each survey site (fig. S4). Transects were run perpendicular to shore across the reef flat to reef crest, thus covering the same area as our coral growth studies (see above) and benthic surveys (see below), while avoiding double-counting the same corals as our benthic surveys. For each coral that intercepted the transect, we identified it to genus and measured its diameter along the transect line to the nearest centimeter as a measurement of colony size (*86*).

#### Statistical analyses

For analyses of coral recruitment and colony size, we first focused on *Acropora* to match data on coral growth and nutrient assimilation and then expanded to look at all corals combined to gain a more general picture of coral recruitment and size distribution. We modeled coral recruitment as a function of seabird versus rat status and a group-level atoll effect following zero-inflated negative binomial distributions, which was appropriate for the high proportion of zeros in our count data. Following (*87*), we log-transformed colony size before analysis (*n* = 463 *Acropora* colonies and 915 all coral colonies). We modeled coral size as a function of rat status and a group-level atoll effect using a skew normal distribution to obtain estimates not only of central tendency (median) but also of SD and skewness of size distributions to represent processes including recruitment rates (*87*). All model fitting and checking procedures were carried out as for hypothesis 1.

### Hypothesis 3: Seabirds speed recovery of coral cover and a return to predisturbance benthic community structure following a major marine heatwave and subsequent mass coral bleaching event

#### Coral and benthic surveys

To measure reef-wide recovery of coral cover and benthic communities, we conducted point-intercept surveys along four 30-m transects at each of 12 islands in April to May 2021 (~6 years after bleaching) (fig. S4). Previous surveys were conducted around the same 12 islands in 2015 (before bleaching) and 10 of these islands in 2018 (~3 years after bleaching), the results of which are published in (*20*). Briefly, we categorized the benthos into broad functional groups: hard coral, soft coral, macroalgae, CCA, pavement (including turf algae), rubble, sand, sponge, and other benthos. We further identified hard coral and macroalgae to genus. *Halimeda* accounted for >99% of all macroalgae, so we kept *Halimeda* as a separate category and reclassified the remaining <1% of macroalgae as “other benthos.” Coral taxonomy is rapidly changing, with recent alterations to genera classifications for a number of corals. Because our surveys only identify corals to genus, and we cannot go back and reclassify previous surveys using updated taxonomy, we grouped corals based on broader taxonomy and functional role following (*86*, *88*).

#### Statistical analyses

We analyzed several metrics of resilience. First, we examined changes to proportional cover of *Acropora* following from our detailed analyses of *Acropora* growth and recruitment. We modeled both *Acropora* and hard coral cover as a function of rat status (seabird and rat), year (2015, 2018, and 2021), and their interaction with island nested within atoll as a group-level effect to account for multiple transects within each island. We modeled proportional cover following a beta distribution because it is bounded between 0 and 1 and works well with percent (or proportional) cover data from ecological datasets (*89*). A small constant was added because of the presence of 0 s in our dataset, following (*89*). All Bayesian model fitting and checking procedures were carried out as for hypothesis 1.

Because trends in coral community composition following disturbance may be dissociated from trends in total hard coral cover (*67*), we also conducted a nonmetric multidimensional scaling analysis (NMDS) using Bray-Curtis dissimilarity to examine any changes in coral community structure and composition. We ran an additional NMDS to examine changes in community structure of broad benthic groups. NMDS stress was 0.15 and 0.16, respectively, indicating acceptable fits. We conducted these analyses using the vegan package (*90*).

We focused on distance of community to prebleaching baseline as a comprehensive measure of the resilience of benthic communities as a whole (*40*, *64*). To calculate the dissimilarity between communities, we calculated Bray-Curtis dissimilarity index within each island for 2018 (3 years after bleaching) and 2021 (6 years after bleaching) compared to 2015 (before bleaching). We then modeled distance to 2015 following a beta distribution as a function of rat status, year, and their interaction with atoll as a group-level effect using a Bayesian model. We chose a beta distribution because Bray-Curtis dissimilarity index is constrained between 0 and 1.

We also used a multivariate Bayesian model to analyze changes in proportional cover of individual benthic groups (hard coral, *Halimeda*, CCA, pavement, and rubble) that together represented 95% of the benthos and were major drivers of community change. As above, we modeled proportional cover following a beta distribution with a small constant added and included the interactive effects of rat versus seabird status and year as explanatory variables with island nested within atoll as group-level effects. All model fitting and checking procedures were carried out as for hypothesis 1.

#### Models of Acropora cover

Because there was a 3-year gap between our surveys, during which time we observed a full recovery of *Acropora* and coral cover (see Results), we used a modeling approach to reconstructing the hidden time series between April 2018 and May 2021 around seabird versus rat islands. We again focused on *Acropora* because it was the dominant component of coral cover in all years, and we had detailed colony growth and recruitment data for this genus. We fitted a hierarchical Bayesian model using JAGS (*91*) and the runjags interface (*92*) in R. We were interested in estimating *C*_*t*,*i*_, the percentage of *Acropora* cover *C* at month *t* around each island *i*. Following other recent studies (*14*, *93*), we used a Gompertz-based modeling approach to describing a Markov process where *C*_*t*,*i*_ evolved at monthly time intervals. An island’s *Acropora* cover during a given month *C*_*i*,*t*+1_ was defined as follows:

$$C_{t+1,i}∼N({C\mu }_{t+1,i},{C\tau }_{t+1,i})\;T(0,100)$$

The precision of this truncated normal distribution *C*τ reflects the biological variation around the expectation *C*μ that we assigned from a gamma distribution with shape and rate parameters of 16 and 6, respectively. The expectation *C*μ of this distribution reflects the Gompertz relationship between “initial” *Acropora* cover *C* (cover in 2018), “maximum” cover *M* (cover in 2021), the rate of change in cover caused by coral growth *G* (hereafter “growth”), and the rate of change of cover caused by coral recruitment *R* (hereafter “recruitment”)

$${C\mu }_{t+1,i}=M_{t,i}\times \exp\left(-\frac{\log(\log\left(\frac{M_{t,i}}{C_{1,i}}\right)}{G_{t,i}}\right)\times \exp(-G_{t,i}\times t)+R_{t,i}$$

We used normal distributions to incorporate ecological variation around the values of maximum *Acropora* cover *M*_*t*,*i*_, growth *G*_*t*,*i*_, and recruitment *R*_*t*,*i*_ at each island

$$M_{t,i}=N({M\mu }_{1,i},M\tau )$$

$$G_{t,i}=N({G\mu }_{1,i},G\tau )$$

$$R_{t,i}=N({R\mu }_{1,i},R\tau )$$

Here, *M*μ_1,*i*_ was the mean percentage cover of *Acropora* at each island in 2021. The parameter for growth at each island *G*μ_1,*i*_ was calculated as the proportion of the average coral growth rate (11.2 cm^2^/month at seabird islands and 4.9 cm^2^/month at rat islands) multiplied by the average density of *Acropora* individuals across all islands (3.6 per m^2^). The parameter for recruitment *R*μ_1,*i*_ at each island was assumed to be 0.173 and 0.227 cm^2^/month at seabird and rat islands, respectively, due to average recruitment of *Acropora* colonies of <5 cm being 0.52 and 0.68 colonies/m^2^ per year, respectively. To account for uncertainty in these parameters, we assigned gamma priors (expressed in terms of shape and rate) to *M*τ, *G*τ, and *R*τ. The precision of the normal distribution of final *Acropora* cover (*M*τ) had a mean of 25 and an SD of 5, i.e., gamma(25,1), and the precision of growth (*G*τ) and recruitment *R*τ had means of 1111 and a SD of 25, i.e., gamma(1975,1.77). We ran our model for four chains, with 3000 iterations and a burn-in of 1000 iterations per chain and calculated when *Acropora* cover reached 90%.
