# Supplementary material for: Seabirds boost coral reef resilience
Source: Sci Adv. 2023 Dec 6;9(49):eadj0390. doi: 10.1126/sciadv.adj0390 (PMC10699780; doi:10.1126/sciadv.adj0390)
Supplement: Supplementary file 1 — Figs. S1 to S4 [file sciadv.adj0390_sm.pdf]

Science Advances
AAAS

Supplementary Materials for

Seabirds boost coral reef resilience

Cassandra E. Benkwitt *et al.*

Corresponding author: Cassandra E. Benkwitt, c.benkwitt@lancaster.ac.uk

Sci. Adv. **9**, eadj0390 (2023)
DOI: 10.1126/sciadv.adj0390

This PDF file includes:

Figs. S1 to S4

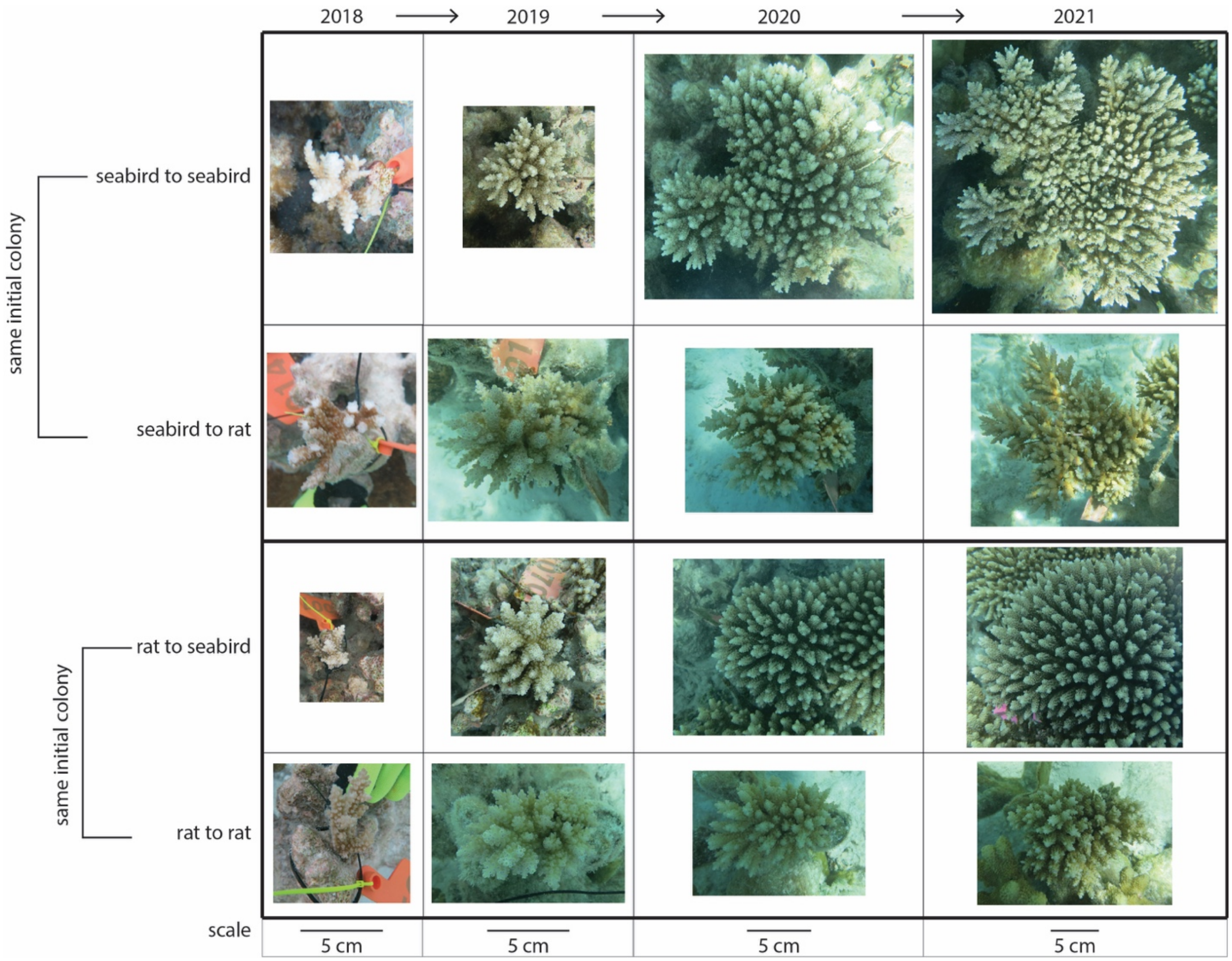

Fig. S1. Images of coral colonies reciprocally transplanted between rat-free islands with abundant seabirds ('seabird islands') and rat-infested islands with few seabirds ('rat islands') from 2018 – 2021. The top two rows illustrate growth trajectories of fragments from the same colony originating from a seabird island transplanted to either the same seabird island or rat island. Similarly, the next two rows illustrate growth trajectories of fragments from the same colony originating from a rat island transplanted to a seabird island or the same rat island. Scales are consistent within each year, but differ across years to allow visualization of all colonies on the same page.

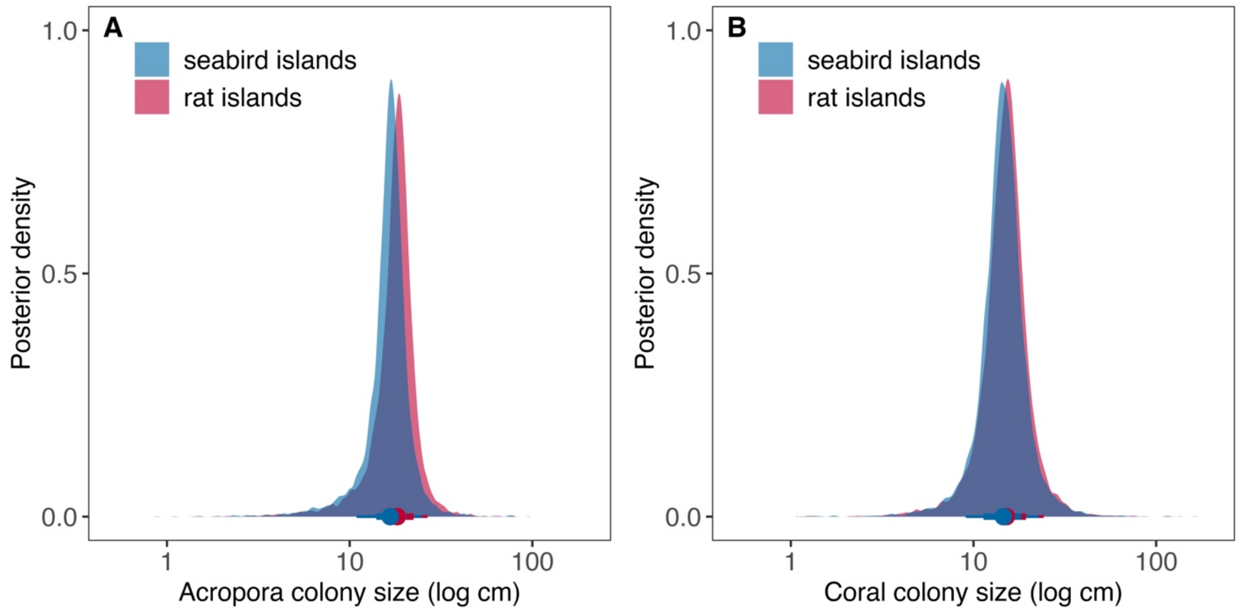

Fig. S2. Posterior density distributions for coral colony size in 2021 around rat-free islands with abundant seabirds ('seabird islands') versus rat-infested islands with few seabirds ('rat islands') of (A) *Acropora* corals only, and (B) all genera combined. Points represent median estimates and lines represent 90% and 70% highest posterior density intervals (HPDIs).

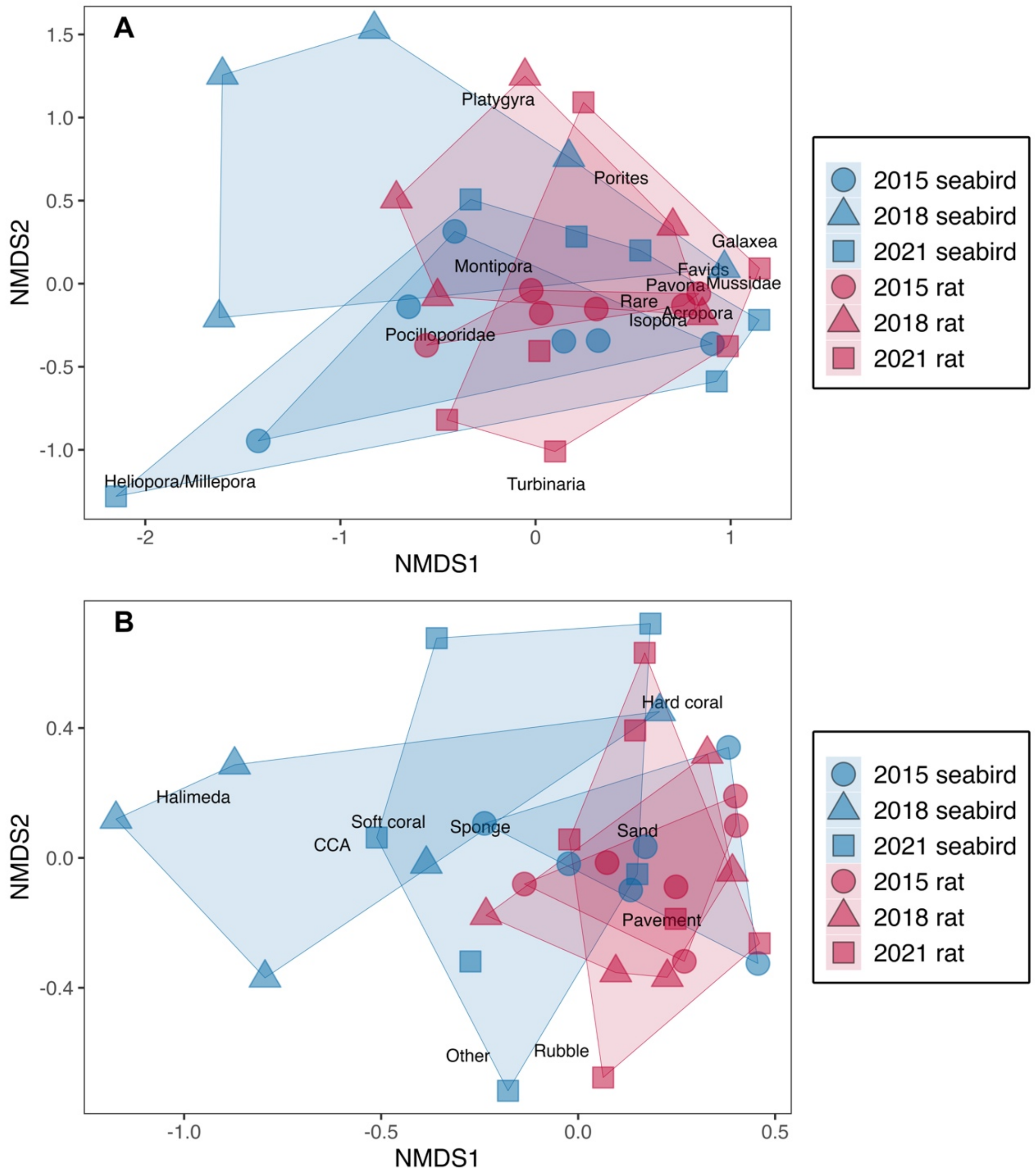

Fig. S3. Nonmetric multi-dimensional scaling analysis for (A) hard corals and (B) overall benthic communities around rat-free islands with abundant seabirds ('seabird islands') versus rat-infested islands with few seabirds ('rat islands') in 2015 (pre-bleaching), 2018 (3 years post-bleaching), and 2021 (6 years post-bleaching). Each point represents one island in one year in species space, with distances among points approximating dissimilarities among communities. Shaded areas represent minimum convex hull polygons for each treatment within each year. Coral groupings are based on broader taxonomy and functional roles, with 'Favids' referring to massive corals formerly classified within Faviidae, now mostly Merulinade, following **(*86*, *88*).** Benthic groupings follow **(*20*).**

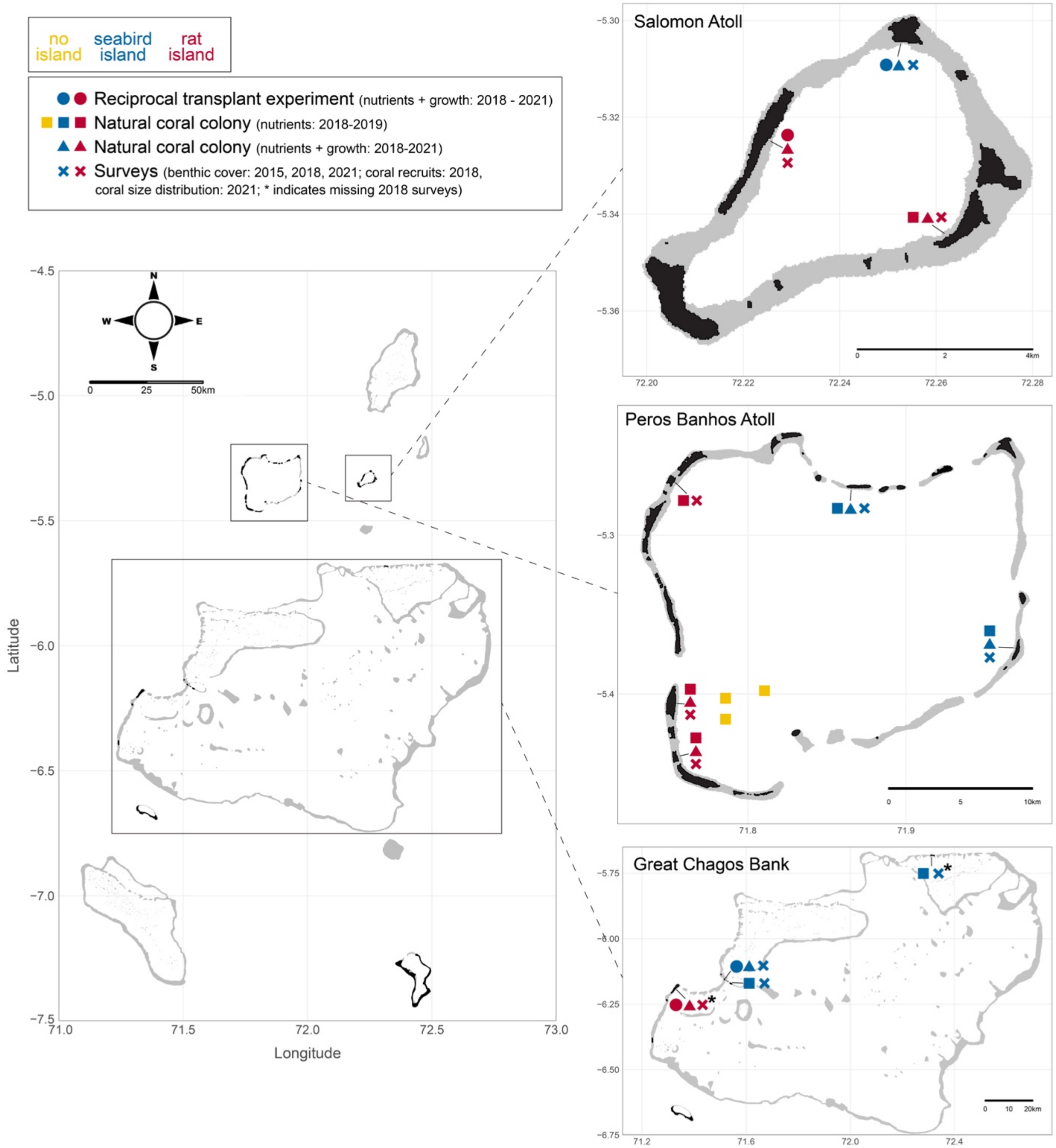

Fig. S4. Map of study region (Chagos Archipelago, Indian Ocean). Symbols indicate where reciprocal transplant experiments (nutrient sampling and growth measurements), natural coral colony studies (nutrient sampling and/or growth measurements), and visual surveys occurred on lagoonal reefs near rat-free islands with abundant seabirds ('seabird island'), rat-infested islands with few seabirds ('rat island'), and control knolls far from islands ('no island').
